# Visual cortical excitability in dementia with Lewy bodies

**DOI:** 10.1192/bjp.bp.114.152736

**Published:** 2016-05

**Authors:** John-Paul Taylor, Michael Firbank, John T. O'Brien

**Affiliations:** **John-Paul Taylor**, MBBS, PhD, MRCPsych, **Michael Firbank**, PhD, Institute of Neuroscience, Newcastle University, Newcastle upon Tyne; **John T. O'Brien**, MD, FRCPsych, Department of Psychiatry, University of Cambridge, Cambridge Biomedical Campus, Cambridge, UK

## Abstract

Alterations in the visual system may underlie visual hallucinations in dementia with Lewy bodies (DLB). However, cortical excitability as measured by transcranial magnetic stimulation (TMS) and functional magnetic resonance imaging (fMRI) activation of lower visual areas (V1–3) to visual stimuli appear normal in DLB. We explored the relationship between TMS-determined phosphene threshold and fMRI-related visual activation and found a positive relationship between the two in controls but a negative one in DLB. This double dissociation suggests a loss of inhibition in the visual system in DLB, which may predispose individuals to visual dysfunction and visual hallucinations.

Dementia with Lewy bodies (DLB) is characterised by complex visual hallucinations. Previously, we used the threshold for eliciting phosphenes with occipital transcranial magnetic stimulation (TMS) as a measure of visual cortical excitability.^1^ Although this threshold correlated with the severity and frequency of visual hallucinations in DLB, overall excitability was similar between DLB and controls, suggesting that early visual areas are functionally intact in DLB. This was supported by our finding of unaltered cortical activity in the early visual system (V1, V2/V3), as measured by blood oxygen level-dependent (BOLD) functional magnetic resonance imaging (fMRI), in DLB.^2^ BOLD haemodynamic response has been suggested to be related to synaptic activity in terms of input and local processing,^3^ as well as to neuronal spiking^4^ arising from sensory stimulation, and thus may represent the activity in both excitatory and inhibitory neuronal populations. In contrast, the neuronal discharge consequent to TMS is likely to be dependent on the relative balance of excitatory and inhibitory activity within the area of stimulation. Therefore a positive relationship between BOLD activity in the early visual system and phosphene threshold may suggest that inhibition outweighs excitation, whereas a negative relationship may indicate the opposite. In this paper we report the interrelationship between fMRI BOLD activity in the early visual system and visual cortical excitability as measured using TMS, using previously published data from patients with DLB and hallucinations and from controls.^1,2^

## Method

Details of participant selection, neuropsychological and neuropsychiatric assessment, fMRI and TMS testing are described fully elsewhere. In the current analysis, we selected participants who had undergone both fMRI and occipital TMS.^1,2^ In brief, 16 participants who met criteria for probable DLB^5^ and who experienced visual hallucinations at least once in the month before TMS/fMRI testing were recruited from a community-dwelling population of patients, along with 19 controls. The study was approved by the local ethics committee. The DLB diagnosis was made independently by two experienced senior clinicians.^5^ Occipital TMS and neuroimaging evaluations were conducted within 2 weeks of each other.

TMS and imaging protocols are described in the online supplementary methods.

## Results

The DLB and control groups were similar in terms of age, gender and visual acuity. Unified Parkinson's Disease Rating Scale (UPDRS)^6^ motor scores were significantly higher and cognitive test and visuoperceptual task scores significantly lower in the DLB group (online Table DS1).

Phosphenes were elicited in a similar proportion of the control (17/19, 90%) and DLB groups (14/17, 82%; Fisher exact test, *P* = 0.66). Across groups, phosphene thresholds were similar (control group: median 70.7%, interquartile range (IQR) = 44.0%; DLB group: median 77.2%, IQR = 36.0%, Mann–Whitney *U*-test 115.5, *P* = 0.23).

There were no differences in V1 or V2/V3 BOLD magnitude between the control and DLB groups for checkerboard stimulus (V1: control group, mean 0.41 (s.d. = 0.36); DLB group, mean 0.57 (s.d. = 0.30), *P* = 0.16. V2/V3 control group: mean 0.68 (s.d. = 0.36); DLB group mean 0.71 (s.d. = 0.29), *P*= 0.83). Similarly, there were no perfusion differences in V1 (ml/100 g/min: control group, mean 33.3 (s.d.= 9.6); DLB group, mean 28.0 (s.d. = 10.5), *P*= 0.20) or V2/V3 (control group, mean 32.1 (s.d. = 8.4), DLB group, mean 27.0 (s.d. = 7.6), *P*= 0.15) between groups. However, there were significant positive correlations between the magnitude of the BOLD response (V1 and V2/V3) to the checkerboard stimulus and phosphene thresholds in the control group and a significant negative relationship in the DLB group in both V1 and V2/V3 (V1: control group, ρ= 0.48, *P*= 0.04; DLB group, ρ= 70.71, *P*= 0.002. V2/V3: control group, ρ= 0.64, *P*= 0.003; DLB group, ρ= 70.57, *P*= 0.02, online Fig. DS1). These relationships remained significant (*P*<0.05) even with the removal of non-responders (i.e. those participants with a phosphene threshold greater than 100% of the stimulator output) except for phosphene threshold *v*. V2/V3 in the DLB group. In TMS responders (i.e. those experiencing phosphenes) BOLD activity was significantly lower in the control group in V1 compared with the DLB group (control group, mean 0.40 (s.d. = 0.37); DLB group, mean 0.65 (s.d. = 0.25), *P*= 0.04).

Differences in the slopes for the control *v*. DLB group for V1 and V2/V3 were highly significant (Fisher *r*-to-*z* transformation: V1, *z*= 3.78, *P*<0.001, V2/V3, *z*= 3.76, *P*<0.001). BOLD activity was significantly lower in V1 and trending lower in V2/V3 in those in the DLB group who were on cholinesterase inhibitors (V1: mean 0.45 (s.d. = 0.24); V2/V3: mean 0.62 (s.d. = 0.10)) compared with those in the DLB group not taking these agents (V1: mean 0.82 (s.d. = 0.28), *P*= 0.02; V2/V3: mean 0.88 (s.d. = 0.33), *P* = 0.10) although phosphene thresholds were similar, as was cerebral perfusion in V1 and V2/V3 between the DLB group on and off cholinesterase inhibitors (phosphene threshold: mean 76.7% (s.d.= 20.2%) *v*. 78.2% (s.d.= 21.1%), *P*= 0.90. Perfusion (ml/100g/min): V1, mean 27.1 (s.d.= 9.7) *v*. 30.4 (s.d.= 13.7), *P* = 0.62; V2/V3, mean 27.4 (s.d.= 7.3) *v*. mean 25.8 (s.d. = 9.4), *P* = 0.73). There was no association between global cognitive function in the DLB group and phosphene threshold, BOLD activity or cerebral perfusion in V1 or V2/V3 (*P*>0.13).

## Discussion

We report a significant positive relationship between phosphene threshold and BOLD activity in early visual areas (V1, V2/V3) in our controls, which suggests that the greater an individual's inherent visual cortical excitability, the smaller the visual cortical activation in response to a simple checkerboard stimulus. This observation implies that individuals with sensitive visual systems need less ‘activation’ to an external stimulus. In contrast, the opposite relationship in DLB infers a breakdown in this dynamic. Assuming similar visual cortical neuronal populations are activated by the checkerboard and TMS, then in the controls a positive relationship between BOLD activity and phosphene threshold implies inhibition must outweigh excitation, whereas in DLB this is reversed. If the BOLD signal represents spiking excitatory neuronal activity,^4^ then the lack of difference between groups in mean BOLD activity suggests a loss of inhibition.

There needs to be critical consideration to our interpretations; our knowledge of the underlying neuronal activity of the BOLD signal is incomplete^3,7^ and the BOLD effect reflects not just neuronal activity but also haemodynamic coupling, which may be altered with neurodegeneration; ideally cerebrovascular reactivity would be assessed via a CO_2_ challenge^8^ or similar paradigm. However, we did not observe any differences in occipital perfusion between the control and DLB group, and similarly, there was no relationship between perfusion and either BOLD response or phosphene threshold. Furthermore, cognitive function in DLB was not associated with phosphene threshold or any of the neuroimaging measures. Thus, although not providing a direct measure of cerebrovascular reactivity, these findings suggest that the early visual system in these participants with DLB was similar to controls.

From the TMS perspective, the origin of phosphene perception may not be co-localised to the visual cortex and thus the relative state of the visual cortex may be less crucial to phosphene perception, although reciprocal connections between early visual and association areas are likely to be important.^9^ In addition, cholinergic function has been implicated in modulating visual attention and associated BOLD fMRI responses;^10^ our observation of differences in BOLD activity between those patients with DLB taking cholinesterase inhibitors *v*. those not, supports this and fits into the broader framework of reported cholinergic receptor changes in DLB^11^ as well as changes in occipital metabolism in individuals with DLB with hallucination amelioration treated with donepezil.^12^

Finally, a loss of inhibitory activity in patients might be expected to decrease phosphene threshold relative to the control group, something which was not observed. However, it is notable that for a range of phosphene thresholds, the overall BOLD response in the DLB group was larger than in the control group, significantly so for V1 after excluding TMS non-responders (whose relative level of visual cortical excitability is unclear). Hence, one could speculate that compensatory mechanisms may be operating in patients with DLB that attempt to ‘normalise’ the balance of excitation/ inhibition in an underactive early visual system, in the face of ‘upstream’ pathology in the visual system thus leading to a maintenance of the phosphene threshold within a normative range and possibly improved bottom-up visual processing, but at the cost of increasing the risk of visual hallucination occurrence, as evidenced by our previous work linking phosphene threshold with visual hallucination frequency and severity.^1^

In summary, our findings of a double dissociation suggest a marked deviance in visual neuronal processing between patients with DLB who hallucinate *v*. healthy controls. Whether this represents a specific loss of inhibitory drive in the visual cortex of people with DLB and what role cholinergic function plays remains to be clarified; neurochemical post-mortem studies or novel spectroscopic imaging assessing for example gamma-aminobutyric acid-ergic tone may be helpful in this regard.
